# Noise-Induced Hearing Loss in Korean Workers: Co-Exposure to Organic Solvents and Heavy Metals in Nationwide Industries

**DOI:** 10.1371/journal.pone.0097538

**Published:** 2014-05-28

**Authors:** Yoon-Hyeong Choi, KyooSang Kim

**Affiliations:** 1 Department of Preventive Medicine, Gachon University Graduate School of Medicine, Incheon, Korea; 2 Occupational Safety and Health Research Institute, Korea Occupational Safety and Health Agency, Incheon, Korea; Harvard University, United States of America

## Abstract

**Background:**

Noise exposure is a well-known contributor to work-related hearing loss. Recent biological evidence suggests that exposure to ototoxic chemicals such as organic solvents and heavy metals may be additional contributors to hearing loss. However, in industrial settings, it is difficult to determine the risks of hearing loss due to these chemicals in workplaces accompanied by excessive noise exposure. A few studies suggest that the effect of noise may be enhanced by ototoxic chemicals. Therefore, this study investigated whether co-exposure to organic solvents and/or heavy metals in the workplace modifies the risk of noise exposure on hearing loss in a background of excessive noise.

**Methods:**

We examined 30,072 workers nationwide in a wide range of industries from the Korea National Occupational Health Surveillance 2009. Data on industry-based exposure (e.g., occupational noise, heavy metals, and organic solvents) and subject-specific health outcomes (e.g., audiometric examination) were collected. Noise was measured as the daily 8-h time-weighted average level. Air conduction hearing thresholds were measured from 0.5 to 6 kHz, and pure-tone averages (PTA) (i.e., means of 2, 3, and 4 kHz) were computed.

**Results:**

In the multivariate linear model, PTA increment with occupational noise were 1.64-fold and 2.15-fold higher in individuals exposed to heavy metals and organic solvents than in unexposed individuals, respectively.

**Conclusion:**

This study provides nationwide evidence that co-exposure to heavy metals and/or organic solvents may exacerbate the effect of noise exposure on hearing loss in workplaces. These findings suggest that workers in industries dealing with heavy metals or organic solvents are susceptible to such risks.

## Introduction

Work-related hearing loss is a critical issue in workplace safety and health. The U.S. National Institute for Occupational Safety and Health (NIOSH) and the occupational safety and health community designated hearing loss as one of the 21 priority research areas in the 21^st^ century [1]. Loud noise is a major risk factor for work-related hearing loss, and audiologists are well aware of the need for monitoring and protecting against noise in the workplace [2]–[4]. In fact, many countries have conducted surveillance and/or compensation programs to monitor workplace noise and identify occupational noise-induced hearing loss [5]–[8].

Evidence accumulated over recent decades suggests that exposure to neurotoxic chemicals is an additional contributor to occupational hearing loss. Some organic solvents, heavy metals, asphyxiants, and pesticides fall within this category and are widely used in many industries and occupations [9]–[11]. Organic solvents such as toluene (C_7_H_8_), styrene (C_8_H_8_), xylene (C_8_H_10_), isopropyl alcohol (C_3_H_8_O), and ethyl benzene (C_8_H_10_) have been widely studied for their ototoxicity. Indeed, animal experiments demonstrate sensorineural hearing loss with cochlear damage as a result of exposure to these chemicals [12]–[16]. Furthermore, several workplace studies suggest that such solvents are associated with an increased risk of hearing loss [17]–[22]. Heavy metals such as cadmium, lead, mercury, and manganese are reported to impair inner ear cells, leading to auditory function disorders in animal studies [23]–[27]. There is further evidence for their ototoxicity in human studies as well [28]–[30].

However, the effects of organic solvents and/or heavy metals on hearing loss in industrial settings are difficult to distinguish from the effect of noise [31]. In fact, many studies in workplaces involving exposure to both solvents and noise commonly assume noise to be the sole contributor to hearing loss; but, they could not observe the effects of solvents despite scientific plausibility that both noise and solvents have biological effects. A possible reason for the null findings of the ototoxic effects of solvents include diluted observation due to the dominant effect of noise; this is because workplaces using these chemicals are likely to be accompanied by excessive noise as well [32], [33]. A few recent studies suggest that the effect of noise can be enhanced by ototoxic chemical exposure [34]–[38].

The Korea National Occupational Health Surveillance (KNOHS), also known as Korean Occupational Medical Examination Program [39], is a nationwide annual surveillance program designed to monitor problematic industries and their workers, including work-related hearing loss. The program includes audiometric testing, industrial-based noise measurement, and assessment of exposure to ototoxic chemicals (e.g., organic solvents and heavy metals).

Thus, the present study estimated the national burdens of occupational noise exposure on work-related hearing loss using nationwide data from various industries from the KNOHS 2009 and evaluated the hypothesis that co-exposure to heavy metals and/or organic solvents in the workplace modifies the risk of occupational noise exposure on hearing loss in a background of excessive noise.

## Methods

### Study population

The final study sample consisted of 30,072 employees aged 18 to 77 years registered in 1,935 industries from the KNOHS 2009; this program was established by the Ministry of Labor of Korea as part of a government program aimed at preventing and compensating for work-related hearing loss.

Data on industry-based exposure and subject-specific health outcomes were collected. The Occupational Safety and Health Research Institute (OSHRI) of the Korea Occupational Safety and Health Agency (KOSHA) compiled a list of all available industries registered with the Korea Ministry of Labor and administered the industries particularly in which the noise exposure level was ≥80 decibels of ambient noise (dBA). These industries were assigned to the KNOHS, which assessed noise exposure levels. All employees who have worked in these industries underwent audiometric examinations and interviews to obtain demographic and physical information. The initial sample included 300,741 participants for primary audiometric examination at limited frequencies (necessarily including 1 kHz) from the KNOHS 2009. From the initial sample, all employees who worked in the industries where the noise level was ≥85 dBA underwent an advanced audiometric examination at various frequencies.

For the present study, we selected participants who had measures in all available frequencies (i.e., 0.5, 1, 2, 3, 4, and 6 kHz); therefore, 64,974 subjects were eligible for inclusion in data analyses. We further excluded participants with missing information for covariate variables (*n* = 33,782), and additional participants were excluded for non-Korean (*n* = 1,120) (see Figure S1). The final analysis included 30,072 participants in the analysis.

### Ethics Statement

The KNOHS is a national dataset collected by the OSHRI that followed the Korean Occupational Safety and Health (OSH) Act [8]. The study protocol was approved by the Occupational Safety and Health Research Institute institutional review board, and written informed consent was provided by study participants.

### Occupational Noise Measures

For each industry, occupational noise exposure assessment was conducted at various onsite spots (1 to 2,109 spots depending on each industry's scale). Exposure was measured as a daily eight-hour time-weighted average (8HR TWA) using a sound level meter (SLM) and was based upon 5 dB exchange rate for calculating noise dose as a function of exposure time and level [40]. The mean noise value from these spot measurements for each industry was computed. All individuals who have worked in the industries with 8HR TWA ≥85 dBA (i.e., noisy industries) were selected to participate in an advanced audiometric examination component.

### Hearing Threshold Examination

An advanced audiometric examination was conducted at various frequencies in a silent room by health technicians trained by a KOSHA-certified audiologist. For standardized measurements, audiometric components, including an audiometer and headphones, and measurement environment followed the guidelines of the Korean Audiometric Quality Control Program [41]. Pure tone air conduction hearing thresholds were obtained for both ears at each frequency over intensity range of −10 to 120 dB and computed as a pure-tone average (PTA) of 2, 3, and 4 kHz following the definition of standard threshold shift of the Occupational Safety and Health Administration. Hearing loss was defined as PTA >25 dB in one or both ears. The details of audiometric examination have been described elsewhere [42]. Hearing thresholds at1 kHz were obtained only in a primary audiometric examination from an initial sampling stage and were not attained in advanced examination in a silent room by trained technicians.

### Ototoxic Chemical Exposures

Non-noise source exposures to organic chemicals and heavy metals were classified based on industries-database compiled by the OSHRI. The OSHRI administered a list of problematic industries exposed to occupational agents (i.e., organic chemicals and heavy metals) among all available industries registered with the Korea Ministry of Labor, similar to a list of problematic noisy industries. Each industry was classed as exposure or not to organic solvents and heavy metals, respectively. Organic solvent exposure was considered for toluene, isopropyl alcohol, and/or xylene (a mixture and individual elements); heavy metal exposure was considered for lead, cadmium, mercury, chrome, and/or manganese (a mixture). Exposure to a mixture was defined as exposure to one or more of the individual elements.

We modeled organic solvent exposure or heavy metal exposure as an exposure to a mixture, because workplaces are not solely exposed to any individual chemical but commonly exposed to a mixture. Instead, sensitivity analyses were examined in all individual organic solvents including toluene, isopropyl alcohol, and xylene. No data regarding individual types of heavy metals were available. Further details are available elsewhere (see File S1).

### Demographic and Potential Risk Variables

Data on demographic and potential risk variables were obtained through physical examinations and extensive interviews. Body mass index (BMI) was computed as weight in kilograms/height in meters squared. Hypertension was classified based on either systolic blood pressure ≥140 mmHg or diastolic blood pressure ≥90 mm Hg at the time of the examination.

### Statistical Analysis

All regression analyses began with univariate analyses to identify outliers and influential points. The statistically significant level was set as *P* values less than 0.05. Linear regression was fit for the association of hearing thresholds in PTA and individual frequencies with occupational noise measures. To identify the influence of potential confounders, we developed a sequence of models —initially crude model; adjusted for age, age-squared, and sex; and further adjusted for BMI and hypertension. We also fit multivariate-adjusted linear regressions in subgroups stratified by age, sex, BMI, and hypertension as well as occupational exposures to organic solvents and heavy metals, in order to evaluate whether these factors modify the association of PTA with occupational noise exposure. Logistic regression was used to estimate odds ratio (ORs) for the hearing loss. All statistical analyses were performed using SAS (version 9.1) and R (2.13.1).

## Results

The KNOHS estimated that 64,974 Korean workers in 7,394 industries were exposed to a daily occupational noise ≥80 dB and that 77% of them had hearing loss (PTA > 25 dB in one or both ears) in 2009 (see Figure S1). Table 1 shows the characteristics of the 30,072 study participants, eligible for the analyses. Their mean ± standard deviation (SD) age was 44.4±9.3 years. Because hearing ability is strongly correlated with age, we computed age-adjusted means for hearing thresholds and other continuous variables. The age-adjusted mean PTA was 28.1±14.7 decibels hearing level (dBHL). The age-adjusted mean of occupational noise exposure was high at 88.6±5.3 dBA, which is unsurprising considering that participants were originally collected from the industries with problematic noise levels. Table 2 shows the occupational noise exposure levels by subject characteristics. Occupational noise exposure was higher for women, older workers, those more exposed to organic chemicals, those less exposed to heavy metals, and those with hypertension and a BMI <25 kg/m^2^ (Table 2).

**Table 1:** General characteristics of study participants (N = 30,072^a^).

Characteristic	Mean^b^ ± SD
Occupational noise^c^	88.6±5.3
Age *(y)*	44.4±9.3
BMI *(kg/m^2^)*	23.6±3.0
Sex [male; *n (%)*]	27,857 (92.6)
Hypertension [*n (%)*]	4,873 (16.2)
Hearing Thresholds *(dBHL)*	
* PTA at 2, 3, 4 Khz* d	28.1±14.7
* 0.5 kHz*	15.6±11.1
* 1 kHz* e	15.8±12.2
* 2 kHz*	18.8±14.3
* 3 kHz*	28.4±17.4
* 4 kHz*	37.3±18.3
* 6 kHz*	41.6±20.3
Hearing Loss^f^ *(PTA>25dBHL, %)*	20461, 68%

^*a*^Participants (N = 33,072) are the individuals having all interest variables in this study: advanced audiometric measurements, age, occupational noise, sex, BMI, and hypertension.

^*b*^Age-adjusted means were presented.

^*c*^Occupational noise (a daily 8-hour time weighted average level in each industry).

^*d*^PTA (pure tone average) of standard threshold at 2, 3, 4 kHz frequencies.

^*e*^Hearing thresholds at 1 kHz were obtained from primary audiometric tests.

^*f*^Hearing Loss (PTA at 2, 3, 4 KHz frequencies > 25 dBHL).

SD, standard deviation.

**Table 2:** Means and 95% confidence intervals of occupational noise exposure by participants characteristics.

Characteristic	No.	Occupational noise (dBA), mean (95% C.I.)		*p-value* a
Age *(year)*				*<0.001*
* 10*–*29*	2356	85.8	(85.6–86.0)	
* 30*–*39*	6995	87.9	(87.8–88.1)	
* 40*–*49*	10062	89.2	(89.1–89.3)	
* ≥50*	10659	90.7	(90.6–90.8)	
Sex				*<0.001*
* Male*	27857	89.0	(89.0–89.1)	
* Female*	2215	90.8	(90.5–91.0)	
Body mass index *(kg/m^2^)*				*<0.001*
* <25*	20991	89.3	(89.3–89.4)	
* ≥25*	9081	88.7	(88.6–88.8)	
Hypertension				*<0.001*
* No*	25199	89.1	(89.0–89.1)	
* Yes*	4873	89.6	(89.5–89.8)	
*Occupational exposure to heavy metals*				*<0.001*
* Non exposure*	27382	89.4	(89.4–89.5)	
* Exposure*	2690	86.1	(85.9–86.3)	
*Occupational exposure to organic solvents*				*<0.001*
* Non exposure*	22164	89.0	(88.9–89.0)	
* Exposure*	7908	89.7	(89.6–89.8)	

CI, confidence interval

^*a*^ *t*-test for binominal groups and *trend-P* for ordinal categorical groups were used.

Table 3 presents the associations between occupational noise exposure and hearing thresholds on PTAs and at each frequency in covariate-adjusted models. In the crude model, occupational noise was significantly associated with elevated hearing thresholds at all frequencies and PTAs (Model A). After adjusting for age and sex, although the associations were attenuated over all frequencies and PTAs, their significance remained, except at 0.5 kHz (Model B). After further adjustment for BMI and hypertension, similar associations between occupational noise and increased hearing thresholds were observed (Model C). In the multivariate-adjusted Model C, the average increase in hearing thresholds in PTAs was 2.35 dBHL (95% confidence interval [CI], 2.05–2.64 dBHL) for an interquartile range (IQR) increase in occupational noise exposure. Further adjustment for occupational exposure to organic solvents and heavy metals did not alter the results (Model D). In all models, the largest rate increase in hearing thresholds with occupational noise exposure was observed at 3 kHz.

**Table 3:** Effect estimates (95% CI) of hearing thresholds (dBHL) with IQR increment in occupational noise exposure (dBA).

Frequency	Model A^a^			Model B^b^			Model C^c^			Model D^d^		
	Per noise IQR^e^			Per noise IQR^e^			Per noise IQR^e^			Per noise IQR^e^		
	Estimate	(95% CI)		Estimate	(95% CI)		Estimate	(95% CI)		Estimate	(95% CI)	
PTA^f^	6.06	(5.73,	6.39)*	2.33	(2.03,	2.62)*	2.35	(2.05,	2.64)*	2.24	(1.94,	2.54)*
0.5 kHz	0.54	(0.32,	0.76)*	−0.10	(−0.33,	0.12)	−0.12	(−0.35,	0.11)	−0.14	(−0.37,	0.09)
1 kHz	1.97	(1.73,	2.22)*	0.62	(0.37,	0.87)*	0.64	(0.39,	0.89)*	0.64	(0.39,	0.89)*
2 kHz	4.22	(3.92,	4.52)*	1.72	(1.43,	2.01)*	1.71	(1.43,	2.00)*	1.65	(1.36,	1.95)*
3 kHz	7.21	(6.82,	7.59)*	2.97	(2.63,	3.32)*	3.01	(2.66,	3.36)*	2.88	(2.52,	3.23)*
4 kHz	6.75	(6.34,	7.16)*	2.29	(1.93,	2.65)*	2.32	(1.96,	2.69)*	2.19	(1.82,	2.56)*
6 kHz	6.90	(6.45,	7.34)*	2.67	(2.26,	3.07)*	2.70	(2.29,	3.11)*	2.54	(2.13,	2.95)*

CI, confidence interval.

^*a*^Model A was a crude model.

^*b*^Model B was adjusted for age, age^2^, sex.

^*c*^Model C: Model B + further adjusted for BMI, hypertension.

^*d*^Model D: Model C + further adjusted for occupational exposures to organic solvents and heavy metals.

^*e*^Hearing Thresholds (dBHL) change per interquartile range (IQR) of occupational noise, 94.26 dBA - 84.74 dBA: 9.52 dBA.

^*f*^PTA (pure tone average at 2, 3, 4 kHz).

*p<0.05.

Table 4 shows the multivariate-adjusted associations of PTAs with occupational noise exposure in groups stratified according to participant status listed in Table 1. There was a significant increase in the associations of PTAs with occupational noise exposure with increasing age (*p_interaction_*  = 0.004) and a marginally stronger association in women than in men (*p_interaction_*  = 0.055).

**Table 4:** Multivariate-adjusted effect estimates (95% CI) of hearing thresholds (dBHL) with IQR increment in occupational noise exposure (dBA), stratified according to participant status.

		Per noise IQR^a^		*p*-value for
Stratification variable	No.	Estimate	(95% CI)	interaction
Overall	30072	2.35	(2.05, 2.64)*	
Age *(year)*				*0.004*
* ≤29*	2356	0.93	(0.17, 1.68)*	
* 30–39*	6995	2.05	(1.51, 2.58)*	
* 40–49*	10062	2.10	(1.58, 2.62)*	
* ≥50*	10659	2.79	(2.23, 3.35)*	
Sex				*0.055*
* Male*	27857	2.23	(1.92, 2.54)*	
* Female*	2215	3.19	(2.28, 4.09)*	
BMI *(kg/m)*				*0.573*
* <25*	20991	2.35	(1.99, 2.70)*	
* ≥25*	9081	2.31	(1.78, 2.84)*	
Hypertension				*0.375*
* No*	25199	2.26	(1.93, 2.58)*	
* Yes*	4873	2.61	(1.91, 3.31)*	

Models were adjusted for age, age^2^, sex, BMI, and hypertension, defined in Model C, Table 3.

CI, confidence interval.

^*a*^PTA (dBHL) change per interquartile range (IQR) of occupational noise, 94.26 dBA - 84.74 dBA: 9.52 dBA.

*p<0.05.

We also evaluated whether the association between occupational noise exposure and hearing outcome is modified by co-exposure to ototoxic chemicals. Table 5 shows the multivariate-adjusted associations of PTA with occupational noise levels in groups stratified according to occupational organic solvent or heavy metal exposure. The PTA increment with the IQR increase in occupational noise was significantly higher in subjects exposed to organic solvents (versus non-exposed subjects, 4.43 dBHL [CI, 3.43*–*5.42] vs. 2.06 dBHL [CI, 1.75–2.37]) as well as in subjects exposed to heavy metals (vs. non-exposed subjects, 3.25 dBHL [CI, 2.58–3.92] vs. 1.98 dBHL [CI, 1.65–2.31]). When individual frequencies were examined, similar higher increments were observed in the organic solvent-exposed group at 3, 4, and 6 kHz as well as in the heavy metal-exposed group at 2 and 3 kHz compared to the respective non-exposed reference groups (see Table S1).

**Table 5:** Multivariate-adjusted effect estimates (95% CI) of hearing thresholds (dBHL) with IQR increment in occupational noise exposure (dBA), stratified according to occupational exposure to ototoxic chemicals.

Stratification variable		Per noise IQR^a^				*p-value* for interaction
	No.	Estimate	(95% CI)			
Overall	30072	2.35	(2.05, 2.64)*			
Heavy metals						*<.001*
* Non exposure*	22164	1.98	(1.65, 2.31)*			
* Exposure*	7908	3.25	(2.58, 3.92)*			
Organic solvents						*<.001*
* Non exposure*	27382	2.06	(1.75, 2.37)*			
* Exposure*	2690	4.43	(3.43, 5.42)*			

Models were adjusted for age, age^2^, sex, BMI, and hypertension, defined in Model C, Table 3.

CI, confidence interval.

^*a*^PTA (dBHL) change per interquartile range (IQR) of occupational noise, 94.26 dBA - 84.74 dBA: 9.52 dBA.

*p<0.05.

Logistic regression models revealed significantly greater odds of hearing loss with occupational noise levels in groups exposed to heavy metals and organic solvents than in the respective non-exposure reference groups (Table S2).

## Discussion

The present study of a nationwide working population demonstrates association of occupational noise exposure with poorer hearing outcomes, and that individuals working in industries that particularly involve exposure to organic solvents and/or heavy metals are susceptible to an increased risk of hearing loss due to occupational noise, suggesting noise–organic solvent interaction and noise–heavy metal interaction.

Work-related hearing loss is one of the most common occupational hazards [1]. Noise is a well-known contributing factor. Thus, many researchers suggest the need to control noise exposure in order to reduce the burden of work-related hearing loss. Although many countries have implemented national programs to monitor and control noise levels in various industries, it is difficult to reduce noise exposure in many industries. Therefore, identifying susceptibility-related factors in conjunction with the avoidance of noise (the major risk factor) is important for effectively preventing occupational noise-induced hearing loss.

Despite biological evidence of the ototoxicity of industrial chemicals, previous studies in occupational settings assume noise to be the sole contributor to hearing loss and observed little effects of ototoxic chemicals. The dominant effect of loud noise in workplaces may explain why such previous studies did not find effects of ototoxic chemicals. Interestingly, 2 recent studies in a general population [43], [44] report adverse effects of lead and cadmium exposure on hearing outcomes not in the group exposed to higher noise levels, but only in those exposed to lower or mid- noise levels; these findings support the notion that the observed risk due to heavy metals may be diluted by the greater risk due to noise. In fact, consistent with previous studies, the present sensitivity analysis stratified according to noise levels revealed that organic solvent exposure adversely affected hearing thresholds in a group exposed to mid-noise levels but found no effect of organic solvent exposure in a group exposed to more noise (Table S3). This suggests that noise exposure is basically the greatest contributing factor to hearing loss while ototoxic chemicals may have additive effects in low-noise environments (that is, when there remains room for additional damage) and should be approached as a modifiable factor in workplaces involving regular high noise levels.

The current study provides evidence of effect modification by the exposure to heavy metals and organic solvents. Individuals exposed to heavy metals and organic solvents had a higher risk of hearing loss in relation to noise than unexposed individuals. The effect sizes of PTA increment with occupational noise were 1.64-fold and 2.15-fold higher in individuals exposed to heavy metals and organic solvents than in unexposed individuals, respectively (3.25 vs. 1.98 dBHL and 4.43 vs. 2.06 dBHL, respectively; see Table 5). Such effect modifications were roughly comparable to estimated effect modification by sex (1.36-fold higher in women [3.19 dBHL] than in men [2.23 dBHL]) and age over 20 years (1.36-fold higher in subjects aged ≥50 years [2.79 dBHL] than in subjects in their 30s [2.05 dBHL]; see Table 4).

Few studies have evaluated the effect modification (i.e., interaction) by organic solvents or heavy metals on the risk of occupational noise towards hearing outcomes. Several small-scale workplace studies compared noise-only exposure cases and noise- and solvent-exposure cases with a control group (i.e., non-exposure). One such study examining 1,117 employees in the yacht, shipping, paint and lacquer, plastics, and footwear industries found an increased risk of hearing loss by co-exposure to solvents; the odds ratios (ORs) of hearing loss were 3.8 and 6.7–21.5 in the cases of noise only, and noise and solvents (i.e., mixtures, styrene, *n*-hexane plus toluene, or styrene plus toluene) compared to a control group, respectively [36]. Another study analyzing the responses of 701 dockyard workers to a questionnaire on exposure revealed that the OR of hearing loss increased approximately 3-fold in the group exposed to only noise but almost 5-fold in the group exposed to both noise and solvent [35]. Although these studies do not provide evidence of multiplicative interaction by solvents on the risk of noise towards hearing loss, they are broadly compatible with the present findings. A previous study of case-control design supported a potential interaction between noise and toluene; the risk of hearing loss was 11 times greater for noise- and toluene-exposure group compared with unexposed group. The estimated effects were larger than the sums of the individual effects [noise-only (4 times) and solvent-only (5 times)] [45]. To our knowledge, this is the first epidemiologic study to evaluate effect modifications by heavy metals on the association between noise and hearing outcomes in the workplace, although there is existing evidence for a main effect of heavy metals on hearing outcomes [4], [28], [46]. Nevertheless, the precise mechanisms underlying the observed effect modifications by heavy metals and organic solvents remain unclear. Previous animal experiments show that solvents such as toluene, styrene, xylene, isopropyl alcohol, and ethyl benzene damage the cochlea (predominantly the supporting and outer hair cells) and provoke irreversible sensorineural hearing loss [12]–[16]. Furthermore, heavy metals such as cadmium, lead, mercury, and manganese induce the impairment (i.e., apoptosis and/or degeneration) of inner ear receptor cells, leading to auditory neuronal function disorders [23]–[27]. These findings provide a possible explanation that heavy metals and/or organic solvents condition the cochlea for damage triggered by oxidative stress produced by noise exposure. In addition, heavy metals and organic solvents are associated with the regulation of intracellular calcium homeostasis [47], [48], and thus, they may play an additional role in auditory hair cell death.

In the present study, the associations between occupational noise and poorer hearing outcomes were stronger, particularly at middle-to-high frequencies. In addition, effect modifications by heavy metals and organic solvents on the associations between noise and hearing outcomes were strong at middle-to-high frequencies. These frequencies are known to be sensitive to noise [49], [50]; therefore, the present observations suggests that heavy metals and organic solvents act as effect modifiers regulating “noise”-induced hearing loss.

This study investigated the effect caused by mixtures of organic solvent(s) or heavy metal(s) on the association between noise and hearing. Indeed, models including an individual organic solvent (e.g., toluene, isopropyl alcohol, and xylene) confirmed similar effect modifications to findings from models including a mixture of organic solvents (data not shown). However, there are no data for different types of heavy metals. Occupational environments do not expose workers solely to any given chemical; instead, workers are commonly exposed to a mixture of chemicals. Furthermore, molecular mechanisms involved in toxicity due to individual solvents (also, metals) are similar. Therefore, this study did not consider individual effects separately.

This study used a hearing outcome with a PTAs of 2, 3, and 4 kHz on a “standard threshold shift” as defined by the Occupational Safety and Health Administration rather than the most commonly used method of PTA definition at speech frequencies (i.e., 0.5, 1, 2, and 4 kHz) as defined by the World Health Organization (WHO). This is because hearing data at 1 kHz were collected only through a primary audiometric test and subsequently without an advanced examination by trained technicians in a silent room. Instead, sensitivity analyses were examined at all individual frequencies including 0.5 and 1 kHz.

The main strength of this study is its large sample population, which included over 30,000 available subjects in industries with problematic noise selected from an initial sample drawn from all industries registered with the Korean Department of Labor. In addition, the present data support the on-site measurement of noise exposure in all industries. Therefore, the present results extend the partial observations of previous epidemiological studies on work-related hearing loss, which are limited to certain occupations and particularly by the difficulty in assessing exposure, to a nationwide scale including a wide range of occupations/industries. In turn, this enabled us to observe co-exposure to noise and ototoxic chemicals in the workplace.

This study has several limitations that should be considered. First, occupational organic solvent and heavy metal exposure are classed as exposure or not, but no data regarding instrumental measured levels were available; thus, the present study may not account for variability in exposure levels of organic solvents and heavy metals. Moreover, noise and other ototoxic exposure were assigned only at the industrial level; exposure variation among different jobs/tasks within the same industry was not accounted for. Second, the present data do not provide information on various potential confounding factors such as smoking or exposure to non-occupational noise (e.g., residential and recreational). Therefore, the true associations of occupational noise and other ototoxic factors with hearing thresholds may be weaker than those reported in the present study. Third, it can be argued that the present findings regarding the effect modification by ototoxic chemicals on the association between noise and hearing loss are due to the correlation between ototoxic chemical exposure and high-level noise. However, the present results demonstrate the risk of hearing loss due to noise is higher in individuals exposed to heavy metals (see Table 5) even if they are exposed to less noise (see Table 2). Finally, although a nationwide representative sample of noise-exposed workers in Korea increased power to our findings, the observed effect modification may not apply to the general population. Because the present data were collected only from industries that have considerable noise levels, and therefore a majority of subjects may have work-related hearing loss and were male, our observations could be different from general populations exposed to low-level noise and who reflect half male and half female. We suggest more research is needed to confirm our evidence using a large sample of general adults.

The present study based on the Korea National Occupational Health Surveillance program estimates that more than 7,000 Korean industries involve occupational daily exposure to levels exceeding 85 dB. These industries include over 60,000 workers, 77% of whom have hearing loss attributable to their work. Indeed, it is difficult to control noise levels for all potentially problematic industries in a country. In addition, noise exposure is common in certain industries and occupations. Therefore, with the need for cost-effective control of noise, the present study provides data for establishing criteria for priority industries to implement monitoring and reduce occupational noise and chemicals to effectively lessen the national burden of work-related hearing loss.

## Conclusion

In conclusion, the present study supports the hypothesis that co-exposure to organic solvents and heavy metals may increase the risk of hearing loss due to noise exposure. The present findings suggest that the risk of noise-induced hearing loss may vary by industry. Furthermore, employees in industries dealing with heavy metals and/or organic solvents are susceptible to such risks, and these industries should prioritize noise and chemicals reduction to prevent work-related hearing loss.

## Supporting Information

Figure S1 **Study participants profile.**
(PDF)

Table S1 Multivariate-adjusted effect estimates (95% CI) of hearing thresholds (dBHL) at individual frequencies with IQR increment in occupational noise exposure (dBA), stratified according to occupational exposure to ototoxic chemicals.
(PDF)

Table S2 Multivariate-adjusted ORs (95% CIs) of hearing loss*^a^* with IQR increment in occupational noise exposure (dBA), stratified according to occupational exposure to ototoxic chemicals.
(PDF)

Table S3 Multivariate-adjusted effect estimates (95% CIs) of hearing thresholds (dBHL) attributable to occupational exposure to ototoxic chemicals, stratified according to noise exposure levels (dBA).
(PDF)

File S1 **Methods: Ototoxic Chemical Exposures, References.**
(PDF)
