# Supplementary figures and images for: Noise-Induced Hearing Loss in Korean Workers: Co-Exposure to Organic Solvents and Heavy Metals in Nationwide Industries
Source: PLoS One. 2014 May 28;9(5):e97538. doi: 10.1371/journal.pone.0097538 (PMC4037174; doi:10.1371/journal.pone.0097538)

**Figure S1.** Study participants profile.

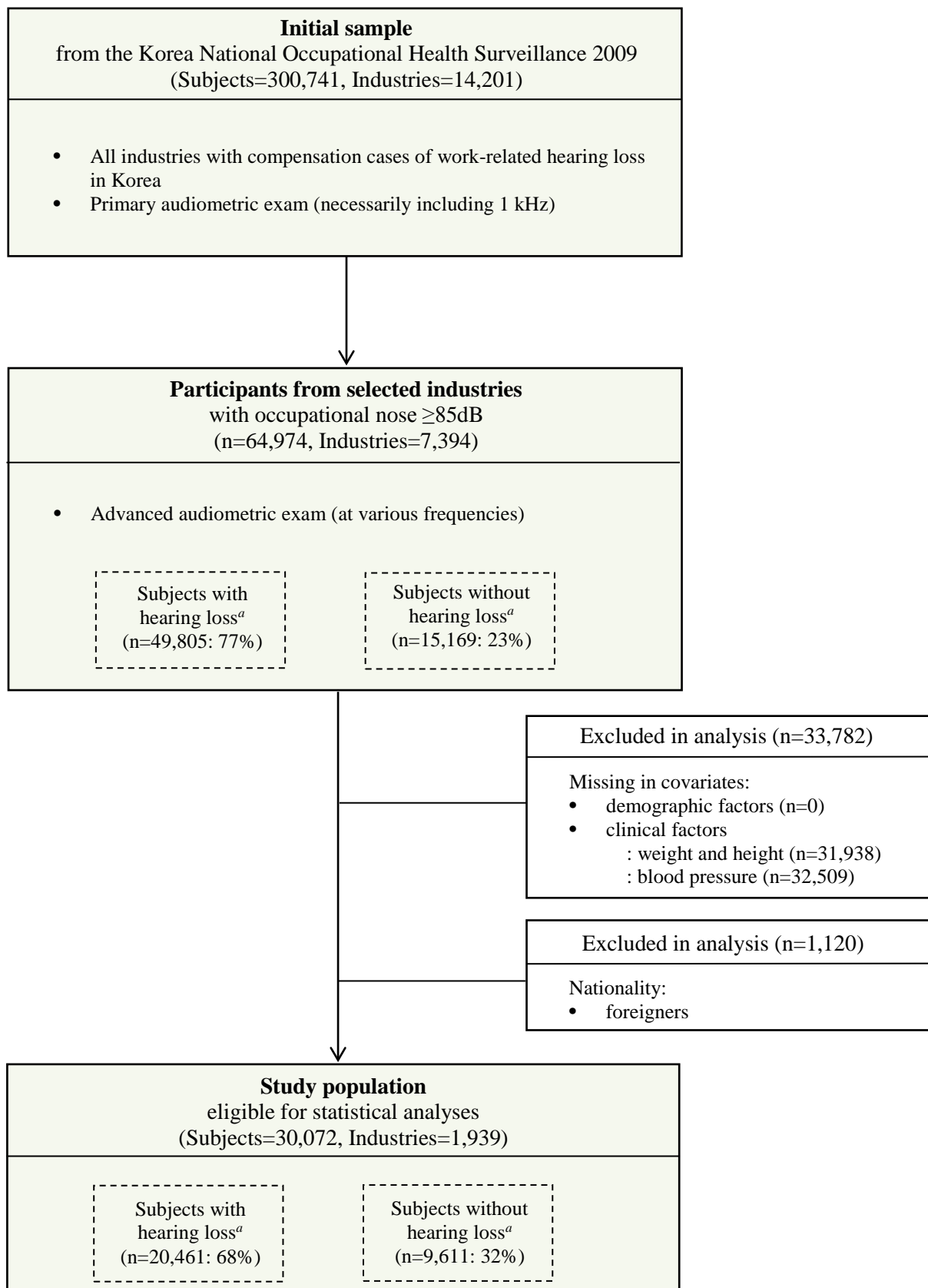

<sup>a</sup>Hearing loss (PTA at 2, 3, 4 kHz frequencies  $>25$  dBHL)

Supplement: Figure S1 — Study participants profile. (PDF) [file pone.0097538.s001.pdf]
