# Supplementary material for: Noise-Induced Hearing Loss in Korean Workers: Co-Exposure to Organic Solvents and Heavy Metals in Nationwide Industries
Source: PLoS One. 2014 May 28;9(5):e97538. doi: 10.1371/journal.pone.0097538 (PMC4037174; doi:10.1371/journal.pone.0097538)
Supplement: Table S1 — Multivariate-adjusted effect estimates (95% CI) of hearing thresholds (dBHL) at individual frequencies with IQR increment in occupational noise exposure (dBA), stratified according to occupational exposure to ototoxic chemicals. (PDF) [file pone.0097538.s002.pdf]

Table S1. Multivariate-adjusted effect estimates (95% CI) of hearing thresholds (dBHL) at individual frequencies with IQR increment in occupational noise exposure (dBA), stratified according to occupational exposure to ototoxic chemicals.

Frequency	Per noise IQR[a]		*p*-value for
Stratification variable	Estimate	(95% CI)	interaction
0.5kHz			
Heavy metals			*<.001*
Non exposure	0.11	(-0.14, 0.36)	
Exposure	-0.90	(-1.43, -0.38)*	
Organic chemicals			*0.185*
Non exposure	-0.23	(-0.47, 0.02)*	
Exposure	0.34	(-0.44, 1.12)	
1kHz[b]			
Heavy metals			*0.283*
Non exposure	0.55	(0.27, 0.82)*	
Exposure	0.89	(0.29, 1.49)*	
Organic chemicals			*0.249*
Non exposure	0.57	(0.31, 0.84)*	
Exposure	1.11	(0.28, 1.95)*	
2kHz			
Heavy metals			*<.001*
Non exposure	1.32	(1.00, 1.63)*	
Exposure	2.82	(2.12, 3.52)*	
Organic chemicals			*0.099*
Non exposure	1.59	(1.29, 1.90)*	
Exposure	2.49	(1.56, 3.42)*	
3kHz			
Heavy metals			*0.002*
Non exposure	2.60	(2.21, 2.99)*	
Exposure	3.98	(3.19, 4.77)*	
Organic chemicals			*0.002*
Non exposure	2.74	(2.37, 3.11)*	
Exposure	4.79	(3.61, 5.97)*	
4kHz			*0.051*
Heavy metals			
Non exposure	2.02	(1.61, 2.44)*	
Exposure	2.95	(2.16, 3.74)*	
Organic chemicals			*<.001*
Non exposure	1.85	(1.47, 2.24)*	
Exposure	6.00	(4.72, 7.29)*	
6kHz			
Heavy metals			*0.120*
Non exposure	2.42	(1.96, 2.88)*	
Exposure	3.24	(2.34, 4.14)*	
Organic chemicals			*<.001*
Non exposure	2.19	(1.76, 2.62)*	
Exposure	6.51	(5.10, 7.92)*	

Models were adjusted for age, age^2, sex, BMI, and hypertension, defined in Model C, Table 3.
CI, confidence interval

[a] PTA (dBHL) change per interquartile range (IQR) of occupational noise, 94.26 dBA - 84.74 dBA : 9.52 dBA

[b] Hearing thresholds at 1 kHz were obtained from primary audiometric tests.

$p<0.05$
