# Supplementary material for: Noise-Induced Hearing Loss in Korean Workers: Co-Exposure to Organic Solvents and Heavy Metals in Nationwide Industries
Source: PLoS One. 2014 May 28;9(5):e97538. doi: 10.1371/journal.pone.0097538 (PMC4037174; doi:10.1371/journal.pone.0097538)
Supplement: Table S2 — Multivariate-adjusted ORs (95% CIs) of hearing lossa with IQR increment in occupational noise exposure (dBA), stratified according to occupational exposure to ototoxic chemicals. (PDF) [file pone.0097538.s003.pdf]

Table S2. Multivariate-adjusted ORs (95% CIs) of hearing loss[a] with IQR increment in occupational noise exposure (dBA), stratified according to occupational exposure to ototoxic chemicals.

Stratification variable	No, with hearing loss/ no. of participants (%)	Per noise IQR[b] OR	(95% CI)	*p*-value for interaction
Overall	20461/30072 (68.0)	1.52	(1.44, 1.60)*	
Heavy metals				*<0.001*
Non exposure	14583/22164 (65.8)	1.46	(1.38, 1.54)*	
Exposure	5878/7908 (74.3)	1.72	(1.52, 1.94)*	
Organic chemicals				*<0.001*
Non exposure	18922/27382 (69.1)	1.43	(1.36, 1.51)*	
Exposure	1539/2690 (57.2)	2.43	(2.01, 2.93)*	

Models were adjusted for age, age^2, sex, BMI, and hypertension, defined in Model C, Table 3.
OR, odd ratio
CI, confidence interval
[a] Hearing Loss (PTA at 2, 3, 4 KHz frequencies > 25 dBHL)
[b] PTA (dBHL) change per interquartile range (IQR) of occupational noise, 94.26 dBA - 84.74 dBA : 9.52 dBA
$*p<0.05$
