# Supplementary material for: Noise-Induced Hearing Loss in Korean Workers: Co-Exposure to Organic Solvents and Heavy Metals in Nationwide Industries
Source: PLoS One. 2014 May 28;9(5):e97538. doi: 10.1371/journal.pone.0097538 (PMC4037174; doi:10.1371/journal.pone.0097538)
Supplement: Table S3 — Multivariate-adjusted effect estimates (95% CIs) of hearing thresholds (dBHL) attributable to occupational exposure to ototoxic chemicals, stratified according to noise exposure levels (dBA). (PDF) [file pone.0097538.s004.pdf]

Table S3. Multivariate-adjusted effect estimates (95% CIs) of hearing thresholds (dBHL) attributable to occupational exposure to ototoxic chemicals, stratified according to noise exposure levels (dBA).

Ototoxic chemicals / Stratification variable	No. of participants	By Ototoxic chemicals[a] Estimate	(95% CI)	*p*-value for interaction
Heavy metals				
Overall	30072	0.88	(0.50, 1.26)*	
Noise[b]				
Low exposure	10010	-0.61	(-1.25, 0.04)	*(Ref)*
Middle exposure	10057	0.38	(-0.30, 1.06)	*0.066*
High exposure	10005	2.42	(1.77, 3.06)*	*<0.001*
Organic solvents				
Overall	30072	0.24	(0.21, 0.27)*	
Noise[b]				
Low exposure	10010	-2.20	(-2.92, -1.48)*	*(Ref)*
Middle exposure	10057	2.29	(0.94, 3.64)*	*<0.001*
High exposure	10005	-1.39	(-2.64, -0.14)*	*0.251*

Models were adjusted for age, age^2, sex, BMI, and hypertension, defined in Model C, Table 3.
CI, confidence interval
[a] PTA (dBHL) change by exposure to heavy metals and organic solvents (vs. non exposure)
[b] Occupational noise exposure tertiles cut-off points: 86.6, 92.69 dBA
$p<0.05$
