# Supplementary material for: Noise-Induced Hearing Loss in Korean Workers: Co-Exposure to Organic Solvents and Heavy Metals in Nationwide Industries
Source: PLoS One. 2014 May 28;9(5):e97538. doi: 10.1371/journal.pone.0097538 (PMC4037174; doi:10.1371/journal.pone.0097538)
Supplement: File S1 — Methods: Ototoxic Chemical Exposures, References. (PDF) [file pone.0097538.s005.pdf]

File S1. Methods: *Ototoxic Chemical Exposures*

Non-noise source exposures to organic chemicals and heavy metals were classified based on industries-database (i.e., KNOHS) compiled by the OSHRI.

The KNOHS is a nationwide annual surveillance program designed to monitor problematic industries and their workers. The KNOHS provides several KNOHS subsets focusing on different kinds of occupational agents. Current study mainly used a KNOHS dataset focusing on occupational noise exposure, and managing occupational noise-related disorders (i.e., hearing loss). They compiled a list of the industries with high-level noise exposure and administered their workers among all available industries registered with the Korea Ministry of Labor. Similarly, KNOHS includes data subsets focusing on occupational heavy metals and organic solvents exposures and managing occupational heavy metals- and organic solvents-related disorders; they compiled lists of the industries with high-level exposures to heavy metals and organic solvents, and administered their workers [1,2]. Organic solvent exposure was considered for toluene, isopropyl alcohol, and/or xylene; heavy metal exposure was considered for lead, cadmium, mercury, chrome, and/or manganese. Each industry was classed as problematic exposure or not to organic solvents (a mixture and individual elements) and heavy metals (a mixture), respectively, and dichotomized variables were ultimately available. Exposure to a mixture was defined as exposure to one or more of the individual elements.

The current study of the KNOHS subset focusing on noise exposure was able to assess exposure-information to heavy metals and organic solvents from two different KNOHS subsets. Note that instrumental measures that accounts for exposure levels were not available in the current study.

We modeled heavy metal exposure or organic solvent exposure as an exposure to a mixture, because workplaces are not solely exposed to any individual chemical but commonly exposed to

a mixture. Instead, sensitivity analyses were examined in all individual organic solvents including toluene, isopropyl alcohol, and xylene. No data regarding individual types of heavy metals were available in the KNOHS.

References

1. OSHRI (2009) Occupational Health Surveillance Standard Methods: Heavy Metals. Incheon, Republich of Korea: The Occupational Safety and Health Research Institute, The Korea Occupational Safety and Health Agency.
2. OSHRI (2009) Occupational Health Surveillance Standard Methods: Organic Solvents. Incheon, Republich of Korea: The Occupational Safety and Health Research Institute, The Korea Occupational Safety and Health Agency.
